# An open-source, modular optoelectrical sensor for on-chip flow characterization in droplet-based microfluidics

**DOI:** 10.1016/j.ohx.2026.e00835

**Published:** 2026-09-08

**Authors:** Daniel Solano, José Isabel Gómez-Quiñones, Sergio Camacho-Leon

**Affiliations:** Tecnologico de Monterrey, School of Engineering and Sciences, Ave. Eugenio Garza Sada 2501 Sur, Col. Tecnologico, Monterrey, Nuevo Leon, 64700, Mexico

**Keywords:** Lab-on-PCB, Embedded systems, Flow-focusing, Droplet sensing, Monodispersity

## Abstract

Droplet-based microfluidics has become a leading technology for Lab-on-Chip applications, from single-cell analysis and nanoparticle synthesis to clinical diagnostics. In these applications, accurate measurement of droplet length, volume, velocity, and monodispersity is critical for reliable operation. Despite recent advances, the routine characterization of these parameters still relies on high-speed cameras, microscopes, and off-chip post-processing, limiting portability, autonomy, and field deployment. To address these limitations, this work presents an open-source, cost-effective, embedded optoelectrical sensor platform built from off-the-shelf components. The platform performs multiparameter droplet characterization on-chip in two sequential firmware stages, with all signal conditioning and analysis executed on an Arduino-Zero microcontroller. The system integrates two custom PCB sensing modules: a light-dependent-resistor (LDR) array for droplet velocity, and a TSL2561 photodiode for length, volume, and monodispersity. Both are illuminated by a dedicated light-source PCB and housed in a 3D-printed alignment enclosure that hosts a replaceable PMMA flow-focusing chip. We provide complete documentation, including build instructions, embedded firmware, and example applications. The resulting modular, equipment-free architecture supports the development of Lab-on-PCB systems for rapid prototyping of autonomous, droplet-based microfluidic devices.

## Graphical abstract

**Figure:**
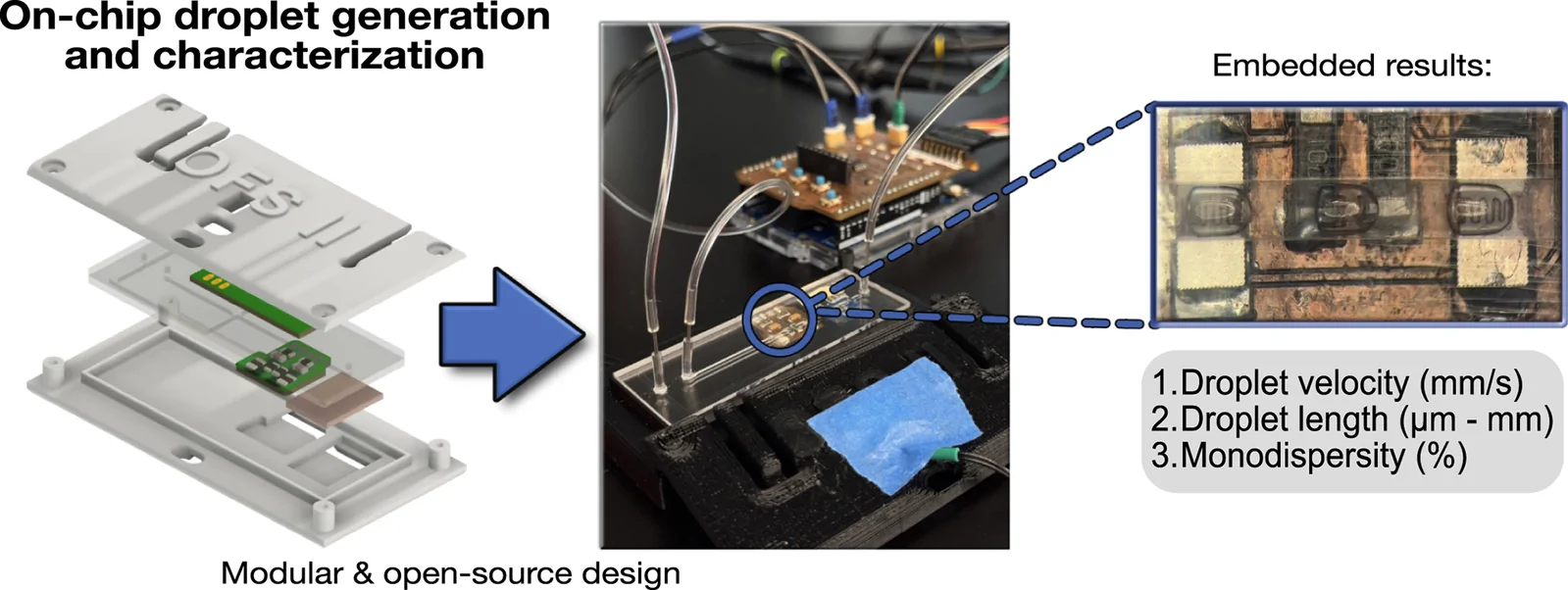

## Specifications table

**Table:** 

Hardware name	Optoelectrical multiparameter droplet-based microfluidic sensor
Subject area	Engineering and material science
Hardware type	Measuring physical properties and in-lab sensors
Closest commercial analog	No commercial analog is available
Open source license	CERN Open Hardware Licence Version 2 – Strongly Reciprocal (CERN-OHL-S v2)
Cost of hardware	Approximately $104 USD (complete module set and processor).
Source file repository	Zenodo, GitHub

## 1 Hardware in context

Droplet-based microfluidics has become one of the most crucial methods for the development of Point-of-Care or Lab-on-a-Chip systems and applications, due to its precision in the generation, manipulation, and processing of small volume droplets dispersed in a continuous fluid [1]. Therefore, the detection and characterization of droplets/slugs becomes critical in applications such as single cell analysis [2], nanoparticle synthesis [3], polymerase chain reaction [4], drug screening [5], enzyme-catalyzed reaction [6], food quality detection [7], among others. The most important parameters to be monitored in droplet-based microfluidics applications are flow rate, droplet velocity, droplet length, and monodispersity (the degree to which droplets are similar in size within a group). Among these, droplet velocity directly affects flow regime stability, monodispersity, mixing efficiency, and downstream processing of the system [1], [8].

Recent works classify the principal detection and characterization methods of droplet-based microfluidics into optical and non-optical categories. Optical techniques include fluorescence, Raman spectroscopy, and absorbance-based approaches, while non-optical methods encompass mass spectrometry (MS), nuclear magnetic resonance spectrometry (NMR), electrical, electrochemical, and magnetic sensing techniques. However, the integration of different detection and characterization technologies into a single device has become a development trend, as it allows higher sensitivity, enhances detection reliability, and broadens the range of detectable analytes [1], [8], [9].

Furthermore, velocity detection and analysis techniques are conventionally performed off-chip, using high-speed cameras together with image processing algorithms, numerical methods, and machine learning techniques [10], [11], [12], [13]. Although these methods yield high-resolution measurements, they require costly optical infrastructure and dedicated computational post-processing pipelines, making them poorly suited for embedded, portable, or resource-limited deployments [8]. As a camera-free on-chip alternative, electrode-based detection has been demonstrated within the channel itself: Lombardo et al. [9] describe a velocity sensor based on an array of electrodes distributed along the microfluidic channel, which allows droplet velocity, size, and content to be determined simultaneously. However, electrode-based methods are inherently sensitive to the ionic composition of the dispersed phase and typically require specialized microfabrication processes, which limits their reproducibility and adaptability across different chip designs. Optoelectrical sensing, in which embedded photodetectors are combined with light sources across the microfluidic channel, offers an alternative on-chip strategy compatible with low-cost polymer chip fabrication and Lab-on-PCB architectures, in which sensing electronics are directly integrated onto the substrate supporting the microfluidic chip [14], [15]. Hassan et al. [16] report a flow cell in which a single LED and photodetector pair aligns with the channel through two apertures separated by a known distance, recovering droplet velocity and droplet size. Signal acquisition and processing run on a host computer, so the measurement remains tied to external software and to the droplet generation setup. Prabatama et al. [17] report a low-cost, open-source optical flow sensor for droplet generators, built around an ESP32 that acquires and processes the phototransistor signal on the sensor board. The prototype reaches a measurement accuracy of 0.76%–13.03% at a total cost of approximately €100, comparable to commercially available flow sensors. The measurement is therefore fully embedded, although the reported output is limited to flow velocity and flow rate, and the results reach the user through a smartphone or web client over an IoT link.

Despite these advances, and to the best of our knowledge, no single low-cost, open-source device reports droplet length, volume, velocity, and monodispersity from fully on-device processing while remaining compatible with standard flow-focusing droplet generation. Table 1 situates the present platform within the existing literature, comparing the measurement capabilities and the level of system integration of related approaches. Beyond the parameters reported, the comparison indicates whether the raw sensor signals can be processed on the measurement hardware itself. This gap is consequential for point-of-care and field-deployable applications, where reliance on high-speed cameras, proprietary software, or external instrumentation represents a fundamental barrier to practical adoption [18], [19].

The modular optoelectrical sensing architecture used in this work was originally reported by Solano and Camacho-León in *Microfluidics and Nanofluidics* [14]. That device aligns a green LED source and a photodetector across a flow-focusing microchannel, so that every droplet crossing the optical path modulates the transmitted light intensity; the resulting lux trace is then segmented by edge detection to recover the droplet boundaries. The study demonstrated a proof-of-concept integration between a flow-focusing droplet generator and off-the-shelf optoelectronic components, and reports the full optical characterization of that sensing principle. However, the droplet-length pipeline was executed off-chip in MATLAB and droplet velocity was extracted using video-based methods, so the system could not operate as a stand-alone instrument.

**Table 1:** Feature-wise comparison of reported droplet characterization approaches.

Work	Sensing principle	Length	Volume	Velocity	Monodispersity	On-device analysis
High-speed imaging [11], [13]	Camera and off-chip image processing	✓	✓	✓	✓	×
Lombardo et al. [9]	Electrode array, electrochemical detection	✓	×	✓	×	×
Hassan et al. [16]	LED and photodetector, dual light path	✓	×	✓	×	×
Prabatama et al. [17]	Optical, flow velocity/flow rate	×	×	✓	×	✓
Solano and Camacho-León [14]	Optoelectrical, single sensing stage	✓	×	×	✓	×
This work	Optoelectrical, two sensing stages	✓	✓	✓	✓	✓

To overcome these limitations, the original proof-of-concept platform was developed into a self-contained embedded device through three main changes: (i) the droplet-length signal-processing pipeline is migrated from the off-chip MATLAB script to firmware running on an Arduino Zero microcontroller, removing the need for host-side post-processing; (ii) an integrated velocity-sensing stage is added to provide the velocity required to convert droplet transit time into length, replacing the previous video-based measurement; and (iii) the two sensing stages are consolidated with the light-source board, alignment enclosure, and user interface into a portable unit that can operate outside a laboratory. The resulting platform reports droplet length, volume, velocity, and monodispersity using on-device processing. It is assembled from low-cost, commercially available components, follows an open-hardware design, and is compatible with standard flow-focusing microfluidic chips. The design was developed in line with WHO ASSURED principles for accessible point-of-care instrumentation [18], [19]. The underlying fluid-dynamics analysis of the velocity and monodispersity relationships is developed in a companion study [20]. Key performance specifications are presented in the Specifications Table; the following sections describe the hardware design, fabrication process, and experimental validation of the proposed sensor.

## 2 Hardware description

**Fig. 1:**
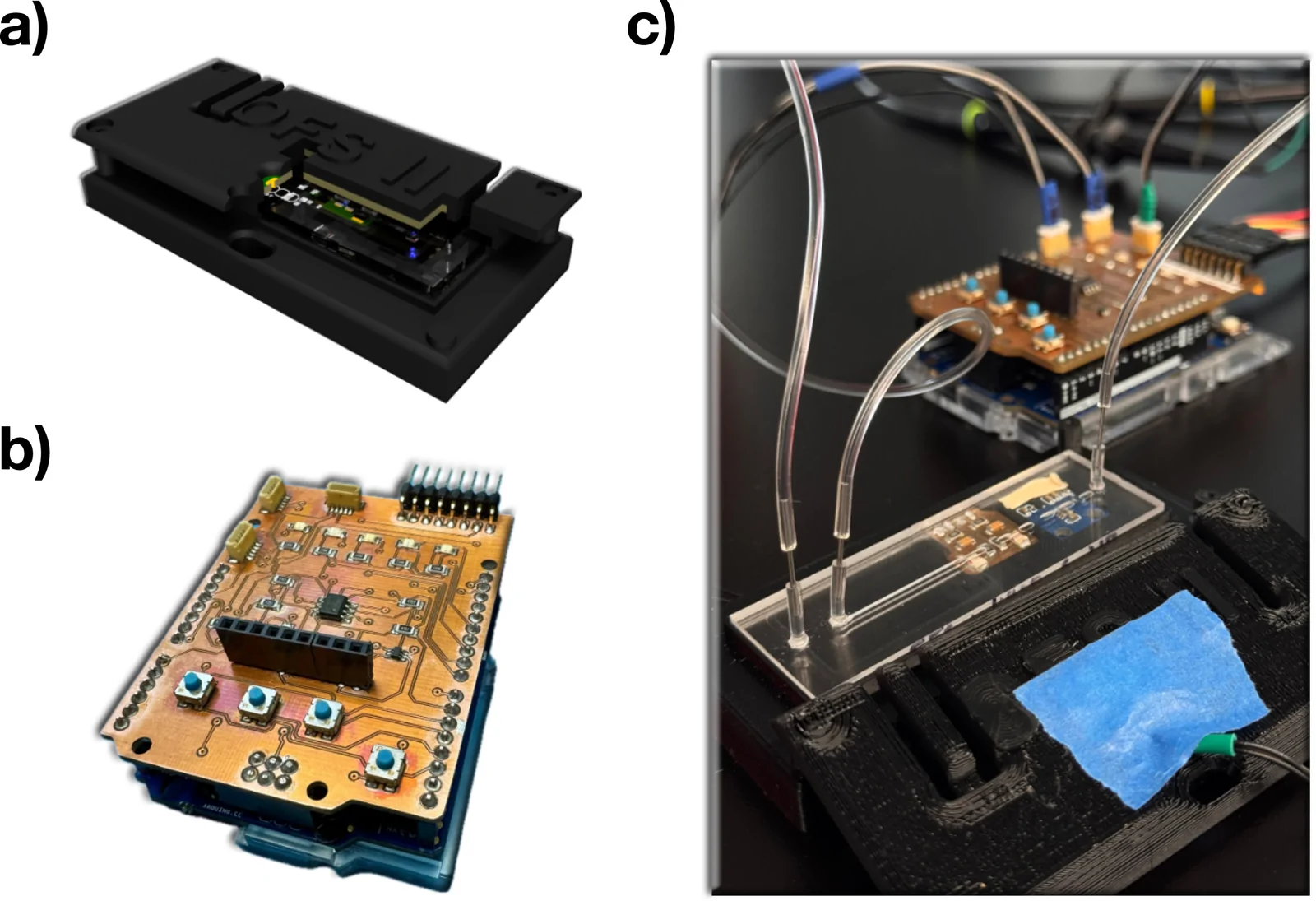
Modular optoelectrical sensor system overview. (a) Assembled main sensor module with two PCB sensing stages, LED light source, and 3D-printed alignment enclosure integrated with a PMMA flow-focusing chip. (b) Arduino-Zero control PCB with push-button controls and LED status indicators. (c) Complete system assembly showing the modular integration of microfluidic chip, PCB sensing modules, and JST interconnects for fully embedded, equipment-free droplet characterization.

The modular optoelectrical sensor system for droplet-based microfluidics is designed to be affordable, modular, and easy to replicate. The system integrates custom-fabricated PCB sensing modules and a 3D-printed alignment enclosure with a poly(methyl methacrylate) (PMMA) flow-focusing droplet generation chip, as illustrated in Fig. 1-(a). All detection and characterization algorithms are embedded on an Arduino-Zero microcontroller paired with a programmable user-interface PCB, which provides LED status indicators and push-button controls for real-time monitoring of sensor outputs (Fig. 1-(b)). Together, these modular elements form a non-invasive, multiparameter embedded system for droplet detection and characterization, as illustrated in Fig. 1-(c).

**Fig. 2:**
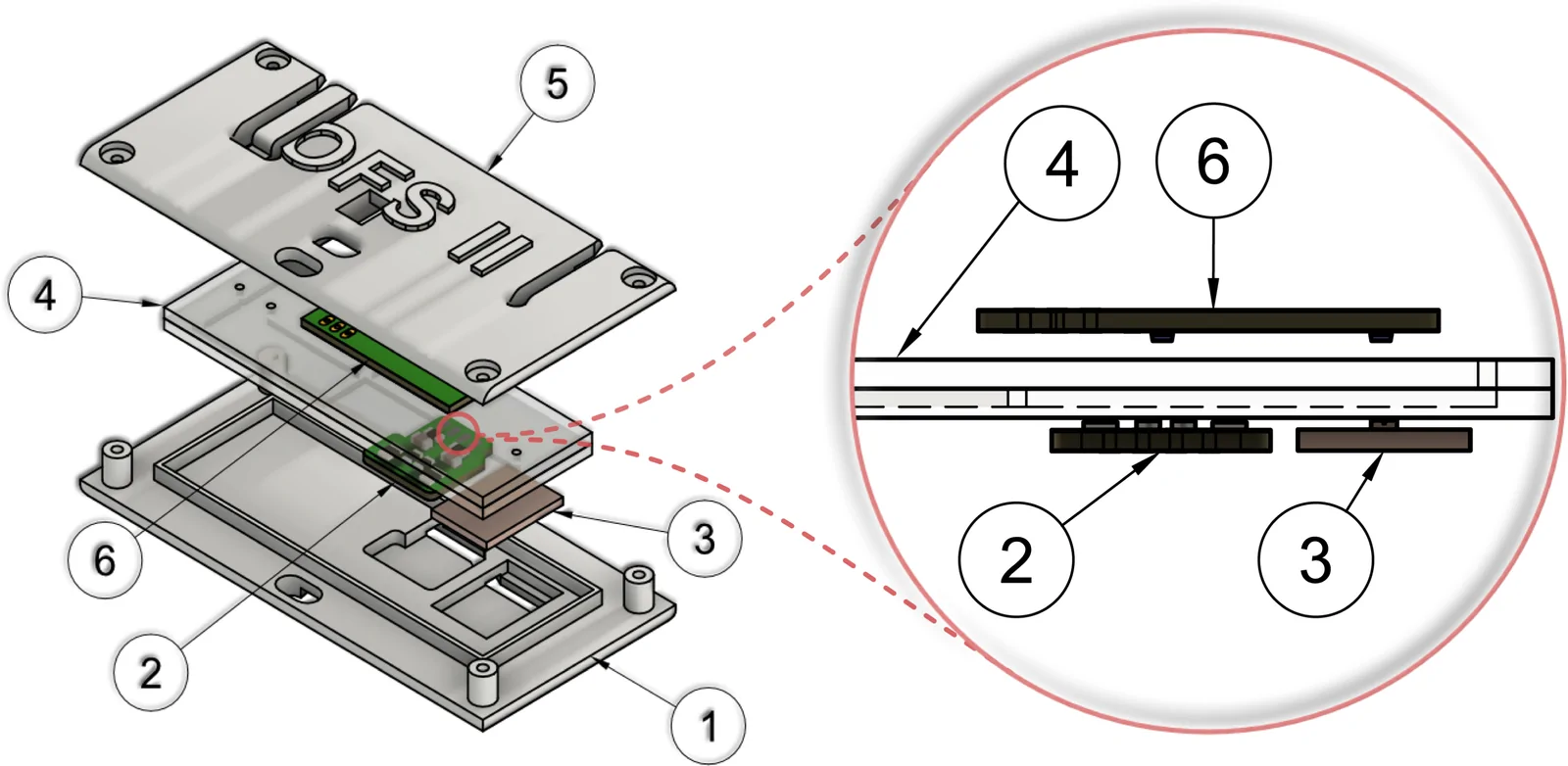
Modular optoelectrical sensor system component detail. (Left) Exploded view of the six main components: (1) base enclosure, (2) droplet velocity sensor (LDR array), (3) TSL2561 sensor PCB, (4) flow-focusing droplet generation chip, (5) top cover, and (6) LED source PCB. (Right) Side-view detail showing the optical alignment between the LED source board (6), the microfluidic channel (4), and the sensing modules (2) and (3).

The main sensor system shown in Fig. 1-(a) comprises six components, as detailed in Fig. 2: two sensing stages (components 2 and 3), a light source PCB (component 6), the flow-focusing droplet generation chip (component 4), and the bottom and top alignment enclosures (components 1 and 5).

The first sensing stage is the droplet velocity sensor board (component 2, Fig. 2). This sensing module uses an array of two surface mount devices (SMD) light dependent resistors (LDRs) separated by a known distance (d=9.88mm), droplet velocity is obtained by the velocity equation (V=d/Δt), in which Δt is the transit time of a droplet between the two LDR sensors. Additionally, to enhance microcontroller performance and a more precise calibration process, a Schmitt-trigger comparator circuit with two digital potentiometers was implemented on the Arduino-Zero PCB as illustrated in Fig. 1. The Schmitt-trigger circuit converts the analog signal of the LDR sensors to digital outputs whenever a droplet is detected and records the detection timestamp using the Arduino-Zero microcontroller.

The second sensing stage performs droplet/slug characterization using a TSL2561 photodiode-based digital ambient light sensor (component 3, Fig. 2). Furthermore, the operational principle of both sensing modules relies on a proper alignment with a light source, obtained using a PCB module with green SMD LEDs aligned with the main microchannel and sensor modules, as shown in the inset of Fig. 2.

The bottom and top alignment enclosures (components 1 and 5, Fig. 2) house all sensor components and shield the sensing modules from external ambient light. The flow-focusing droplet generation chip (component 4, Fig. 2) enables water-in-oil emulsification and is fabricated as an independent, replaceable module.

The embedded firmware implements algorithms for droplet velocity measurement, length estimation, volume calculation, and monodispersity analysis. It also supports data logging and can execute individual characterization routines to allow in-depth study of each sensing stage independently. The open-source character of the design facilitates future adaptations to different applications, supporting scalability and customization to specific experimental requirements. All design files—including PCB Gerber files, Arduino firmware source code, 3D-printed enclosure models, and microfluidic CAD files—are publicly available under the CERN Open Hardware Licence Version 2 – Strongly Reciprocal (CERN-OHL-S v2).

From Fig. 1, Fig. 2, the modular architecture of the electronics, enclosure, and microfluidic systems enables the incorporation of additional sensors and light sources (infrared, phototransistors or photodiodes) for more complex experiments. Furthermore, the modularity also extends to the microfluidic system, which can be fabricated using different geometries, channel sizes, or materials for more elaborate experiments. The only constraint is that the microfluidic device must be optically translucent and conform to standard microscope slide dimensions (75×25mm).

Research applications and benefits:

• **Automated droplet velocity detection:** Enables embedded on-chip droplet velocity measurement without external specialized equipment such as high-speed cameras or microscopes.
• **On-chip droplet characterization:** Enables detection, measurement, and characterization of droplet length, volume, and monodispersity during droplet generation.
• **Non-invasive microfluidic sensing:** The microfluidic droplet generation chip is a separate, replaceable module, enabling reuse across multiple channel geometries and configurations.
• **Customizable multiparameter sensing platform:** The modular Arduino-Zero-based architecture can be expanded with additional sensors or configurations for more elaborate experiments.
• **Cost-effective multiphase microfluidic sensor:** Provides a rapid-prototyping environment for droplet-based microfluidic experiments requiring low-cost, reliable on-chip characterization.
• **Real-time experimental feedback:** Delivers immediate on-chip measurement data, enabling dynamic adjustment and rapid characterization of droplet formation conditions.

## 3 Design files summary

The design files for the current project are divided into three main sections: hardware, Mechanical, and Firmware sections as described in Table 2. The hardware design files provide the complete fabrication data for three printed circuit boards. The velocity sensor PCB Gerber and drill files define a two-LDR sensing configuration with a fixed inter sensor separation of d=9.88mm, which establishes the geometric calibration parameter for time-of-flight velocity estimation. The LED board files describe the PCB layout for the optical sources for both the velocity sensor and the droplet detection/characterization subsystems. The Arduino shield files detail a multi-function interface board incorporating a threshold comparator circuit, digitally-controlled potentiometers for velocity-sensor signal conditioning, a four-button/five-LED operator interface, SPI-connected SD-card storage, and TJS terminal block connectors providing electrical access to all subsystem modules and I/O test points. The mechanical design files provide STEP and STL representations of all physical components: the two-part 3D-printed enclosure (top and bottom shells) designed to house and align the full assembly; dimensional models of each PCB module (Arduino shield, LED board, TSL2561 sensor, and velocity sensor) for positional reference and enclosure fit verification; and a 3D representation of the microfluidic chip geometry, noting that microfluidic chip fabrication is performed via CNC milling rather than additive manufacturing, for this example. The firmware files are organized into two PlatformIO projects: the main C++ source files implementing the droplet detection and characterization routine, encompassing TSL2561 optical signal acquisition, event classification, and SPI-based SD-card data logging; and the C++ source files (.cpp/.h) constituting the velocity sensor firmware, which incorporates the algorithm for computing instantaneous droplet velocity from the time-of-flight signal pair generated by the two-LDR sensor board.

**Table 2:** Design files summary.

Design filename	File type	Open source license	Location of the file
Hardware			

Velocity sensor	Gerber & drill files	CERN-OHL-S v2	design/hardware/velocity_sensor
Light source (LEDs) board	Gerber & drill files	CERN-OHL-S v2	design/hardware/leds_board
Arduino shield	Gerber & drill files	CERN-OHL-S v2	design/hardware/arduino_shield

Mechanical			

Top enclosure	STEP/STL	CERN-OHL-S v2	design/mechanical/top_enclosure
Bottom enclosure	STEP/STL	CERN-OHL-S v2	design/mechanical/bottom_enclosure
Arduino shield	STEP/STL	CERN-OHL-S v2	design/mechanical/arduino_shield
Light source (LEDs) board	STEP/STL	CERN-OHL-S v2	design/mechanical/leds_board
Microfluidic device	STEP/STL	CERN-OHL-S v2	design/mechanical/microfluidic_device
TSL2561 sensor	STEP/STL	CERN-OHL-S v2	design/mechanical/tsl2561_sensor
Velocity sensor	STEP/STL	CERN-OHL-S v2	design/mechanical/velocity_sensor

Firmware			

Droplet detection & characterization – main firmware	C++ source files (.cpp, .h, platformio.ini)	CERN-OHL-S v2	firmware/optical_flow_sensor

Velocity sensor – firmware	C++ source files (.cpp, .h, platformio.ini)	CERN-OHL-S v2	firmware/velocity_sensor

## 4 Bill of materials summary

The off-the-shelf electronic boards required to assemble the sensor system are listed in Table 3. These include the Arduino Zero microcontroller, which serves as the central processing unit of the system, a TSL2561 ambient light sensor for droplet optical characterization, and a Micro SD card breakout board with its corresponding storage medium for onboard data logging. The final cost of these commercial modules amounts to 67.78 USD.

The discrete electronic components required for fabricating the printed circuit boards (PCBs), components 6 and 2 from Fig. 2 along with Fig. 1-b, are enumerated in Table 4. Passive components include decoupling capacitors and resistors in 1206 SMD package format, while active devices comprise the LM393 dual comparator and two MCP4018T digitally controlled potentiometers for velocity-sensor signal conditioning. Additional items include JST connectors, pin headers, LED indicators, tactile push buttons, light-dependent resistors, and the copper-clad substrate from which the PCBs are chemically etched. The total material cost for PCB fabrication amounts to 34.49 USD.

**Table 3:** External hardware boards.

Designator	Component	Number	Cost per unit (USD)	Total cost (USD)	Source of materials	Material type
Arduino Zero	Microcontroller	1	$48.80	$48.80	Arduino.cc	Electronics
TSL2561	Light sensor	1	$5.98	$5.98	Amazon	Electronics
Micro SD SPI or SDIO card breakout board	Micro SD card reader	1	$3.50	$3.50	Adafruit	Electronics
Micro SD card	Micro Memory Card 8 GB	1	$9.50	$9.50	Amazon	Electronics

**Total**				**$67.78**		

The structural and microfluidic components of the assembly are listed in Table 5. The two-part housing (top and bottom enclosure shells), which corresponds to component 5 and 1 from Fig. 2, is produced by 3D printing using PLA filament, with per-unit costs estimated from filament mass consumption. The microfluidic chip (component 4 of Fig. 2) is fabricated by CNC milling PMMA sheets bonded using isopropyl alcohol. The combined material cost of these components amounts to 1.49 USD.

**Table 4:** Electronic components for PCB fabrication.

Designator	Component	Number	Cost per unit (USD)	Total cost (USD)	Source of materials	Material type
Comparator:C1, C2; Vel:C1, C2	0.1 μF Capacitor - 1206	4	$0.51	$2.04	Mouser	Electronics
Comparator:LM393	LM393	1	$0.34	$0.34	Mouser	Electronics
Comparator:POT_S1, POT_S2	MCP4018T	2	$0.68	$1.36	Mouser	Electronics
Comparator:R_HYST1, R_HYST2	480K Resistor 1206	2	$0.10	$0.20	Mouser	Electronics
Comparator:R1, R2, R3, R4	4.7K Resistor 1206	4	$0.10	$0.40	Mouser	Electronics

Comparator:TEST_PORT; Sd1:SD_READER	40× Male Pin Header	2	$0.89	$1.78	Mouser	Electronics

Jst1:TSL2561; Jst2:VELSENSOR; Jst3:LEDBOARD	JST_BM05B	3	$0.63	$1.89	Mouser	Electronics

Ui1:D1, D5; Main; VS	Green 1206 LED	4	$0.25	$1.00	Mouser	Electronics
Ui1:D2	Red 1206 LED - Generic	1	$0.20	$0.20	Mouser	Electronics
Ui1:D3	Yellow 1206 LED - Generic	1	$0.21	$0.21	Mouser	Electronics
Ui1:D4	Blue 1206 LED - Generic	1	$0.27	$0.27	Mouser	Electronics
Ui1:R1, R2, R3, R4, R5; Main; VS	220 Resistor 1206	7	$0.14	$0.98	Mouser	Electronics
Vel:R1, R2, R3, R4	10K Resistor 1206	4	$0.10	$0.40	Mouser	Electronics
Copper Clad	6 × 4 in clad	1	$15.75	$15.75	Digi-Key	PCB board
S1, S2	LDR	2	$0.705	$1.41	AliExpress	Electronics
Ui1:S1, S2, S3, S4	Push button	4	$0.87	$3.48	Digi-Key	Electronics

**Total**				**$31.71**		

**Table 5:** Enclosure and microfluidics components.

Designator	Component	Number	Cost per unit (USD)	Total cost (USD)	Source of materials	Material type
Top enclosure	5	1	$0.22 (17.15 gr)	$0.22	BambuLab	PLA
Bottom enclosure	1	1	$0.27 (20.42 gr)	$0.27	BambuLab	PLA
Microfluidics	4	1	$1.00	$1.00	Amazon	PMMA

**Total**				**$1.49**		

## 5 Build instructions

The fabrication of the proposed system followed the ASSURED criteria for accessible point-of-care instrumentation [18], [19], prioritizing affordable, reproducible, and easy-to-fabricate manufacturing techniques for a portable, easy-to-use device. Accordingly, the construction process was organized into four independent stages: (i) printed circuit board fabrication and assembly, (ii) flow-focusing microfluidic chip fabrication, (iii) enclosure manufacturing, and (iv) firmware deployment and final system assembly. All design files referenced in the following sections are itemized in Table 2, and the corresponding component parts are detailed in the bill of materials tables (Table 3, Table 4, Table 5).

### 5.1 Printed circuit board fabrication

**Fig. 3:**
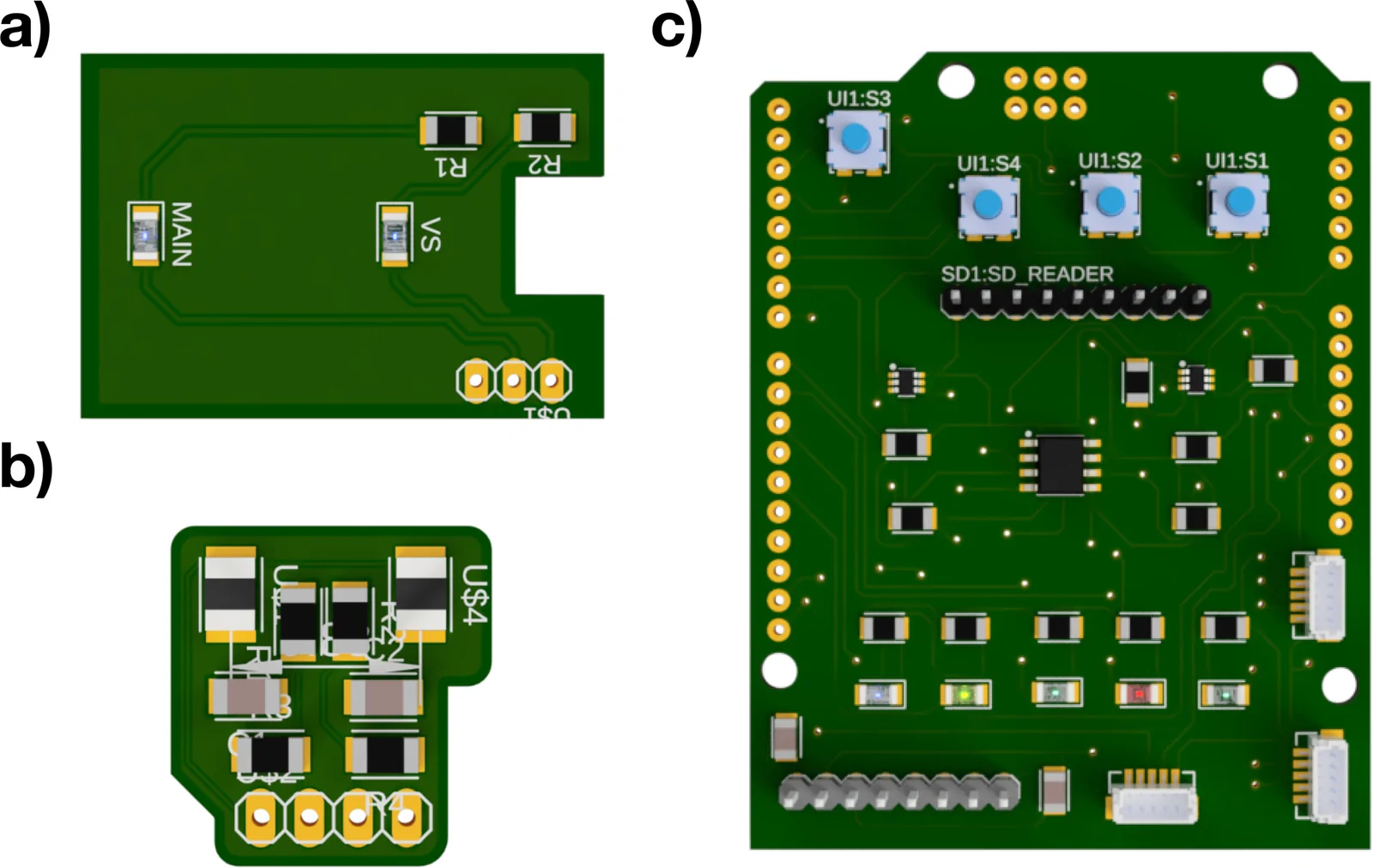
Fabricated printed circuit boards (PCBs) for the modular sensor system. (a) Light source board carrying two green 1206 SMD LEDs for illumination of the droplet detection and characterization stages. (b) Velocity sensor board integrating two surface-mount light-dependent resistors (LDRs) at a center-to-center separation of d=9.88mm. (c) Arduino-Zero shield providing the user interface (four push-buttons and five LED indicators), data logging via Micro-SD card, and the signal conditioning circuitry for the velocity sensor and TSL2561 modules.

The sensor system integrates three printed circuit boards (PCBs): the light source board, the velocity sensor board, and the Arduino-Zero shield, as described in Fig. 3-(a), (b), and (c), respectively. All PCBs were designed and fabricated using an LPKF ProtoMat S63 circuit board plotter, following the procedure previously reported in [14]. The light source and velocity sensor boards were fabricated on single-layer FR4 copper-clad laminate, while the Arduino-Zero shield was fabricated on double-layer FR4 copper-clad laminate to accommodate all circuit elements within the Arduino Zero mechanical footprint.

The light source board, described in Fig. 3-(a), contains two green 1206 SMD LEDs driven through a 220 Ω current-limiting resistor at 3.3 V. The (MAIN) LED is positioned directly above the TSL2561 light sensor, as illustrated in the inset of Fig. 2. The (VS) LED is positioned along the midline between the two light-dependent resistors (LDRs) of the velocity sensor, as shown in the inset of Fig. 2.

The velocity sensor board, described in Fig. 3-(b), hosts the two 1206 SMD LDRs (S1 and S2 in Table 4) at a fixed center-to-center separation of d=9.88mm, which defines the geometric calibration parameter of the time-of-flight velocity estimator described in [20].

The Arduino-Zero shield, described in Fig. 3-(c), integrates the Schmitt-trigger comparator stage (LM393 plus the two MCP4018T digital potentiometers for precise calibration), the four-button and five-LED user interface, the SPI Micro-SD card breakout connector, and the JST terminal blocks providing electrical access to the velocity sensor and TSL2561 sensor modules. As an alternative to in-house fabrication with the LPKF plotter, the provided Gerber files for all three boards can be submitted directly to a commercial PCB house; all designs use hand-solderable SMD packages, remaining compatible with hobbyist-grade fabrication tolerances.

### 5.2 Flow-focusing microfluidic chip fabrication

The flow-focusing droplet generator was fabricated from two translucent poly(methyl methacrylate) (PMMA) CNC milled layers and bonded by solvent-assisted thermal process, following the protocol previously reported in [14], [21]. PMMA was selected for its clinically established biocompatibility, high optical transparency in the 500–600 nm range compatible with the green LED illumination, ease of micro-machining, and low cost relative to glass or silicon (Table 5) [22], [23], [24].

The bottom layer was machined with the flow-focusing design geometry and dimensions, which in this example uses a main channel width of w=1.40mm and depth of h=1.40mm. The top PMMA layer holds the inlet and outlet through-holes for the Tygon tubing fittings. The two machined layers were cleaned with isopropyl alcohol (IPA) and then dried under a pressurized nitrogen stream to remove machining debris and surface contaminants.

The bonding process started with the deposition of a thin film of IPA on the top PMMA layer to act as a solvent welding agent. The two layers were then aligned and transferred to the LPKF MultiPress S, and pressed at 70 °C under a uniformly distributed load of 80 N cm^−2^ for 10 min.

**Fig. 4:**
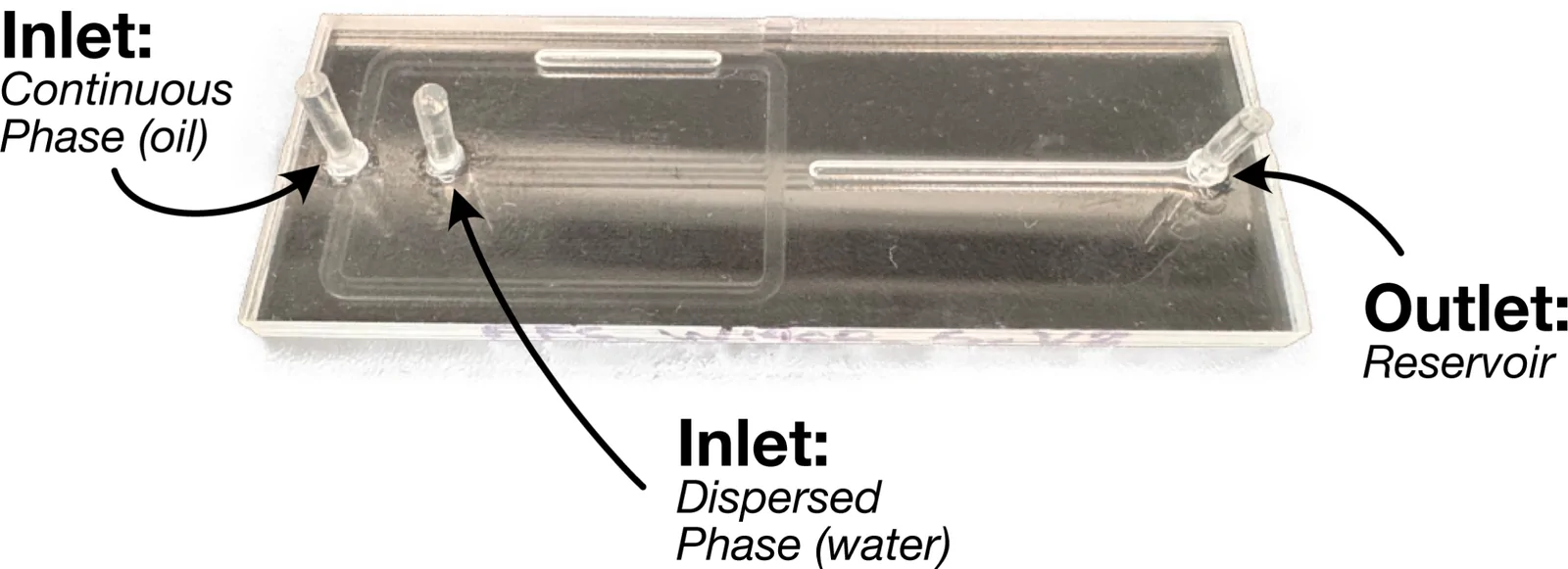
Flow-focusing droplet generation chip. The device was fabricated from two poly(methyl methacrylate) (PMMA) layers machined by CNC milling and bonded by a solvent-assisted thermal process. The main channel width and depth are w=h=1.40mm, and the final chip dimensions follow a standard microscope slide footprint (76.00×26.00×4.46mm).

The final bonded chip measures 76.00×26.00×4.46mm for a final flow-focusing device, as described in Fig. 4, following a standard microscope slide footprint.

The same protocol can be reused to fabricate microfluidic chips with alternative channel widths (e.g., a 700μm variant was successfully produced in [14]) without modifying any other system component, owing to the modular replaceable-chip design.

### 5.3 Enclosure fabrication

The two-part alignment enclosure (top cover and base) was manufactured by 3D printing using opaque black PLA filament on a Zortrax M300 Plus printer. The opaque PLA selection isolates the optical path from external ambient light, essential for reliable threshold detection at both the LDR velocity sensor and the TSL2561 characterization stage. Additionally, it provides the crucial alignment between light sensors and light sources described in Fig. 2. STEP and STL files for both shells are provided in Table 2, and the corresponding material costs are summarized in Table 5.

The enclosure architecture follows the modular plug-and-play philosophy introduced in [14]: the top shell hosts the LED source board and seals the optical path against external illumination, while the TSL2561 sensor PCB, the velocity sensor PCB, and the microfluidic device are accommodated in dedicated chambers to consolidate a proper alignment.

### 5.4 Final system assembly

**Fig. 5:**
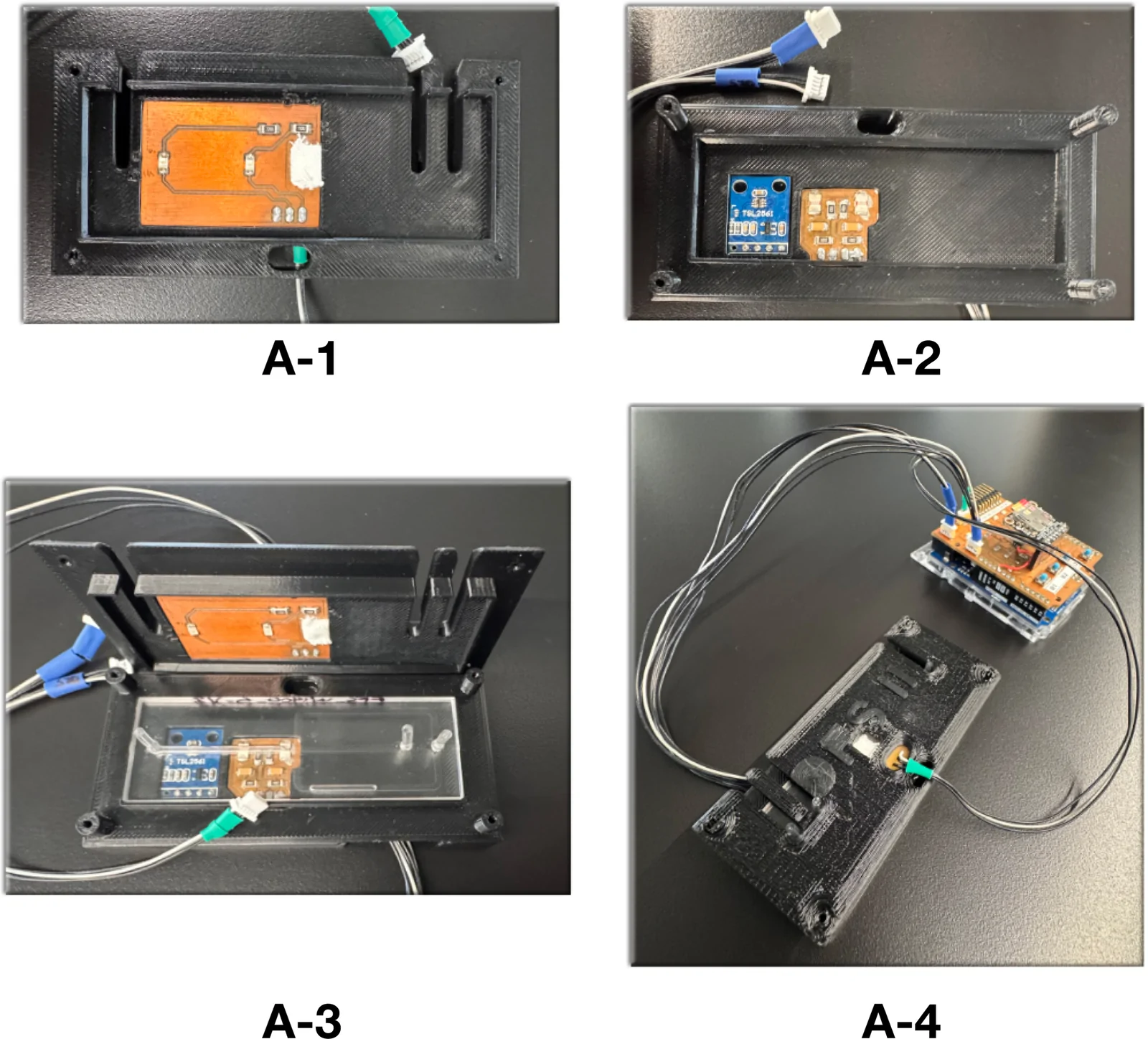
Step-by-step assembly of the modular optoelectrical sensor system. (A1) Placement of the LED source board (component 6) into the dedicated chamber of the top shell and routing of its JST cable. (A2) Mounting of the TSL2561 sensor board (component 3) and the velocity sensor board (component 2) onto the fixing posts of the base shell. (A3) Insertion of the bonded PMMA microfluidic chip (component 4) into the base shell chamber with inlet and outlet ports facing upwards. (A4) Connection of JST cables to the Arduino-Zero shield, seating of the Micro-SD card, and closure of the top cover (component 5) to seal the optical path.

Once the PCBs were populated, the microfluidic chip was bonded and leak-tested, and the enclosure was printed, the complete sensor system was assembled following the sequence summarized below and illustrated in Fig. 1, Fig. 2, Fig. 5.

1. Placement of the LED source board (component 6 in Fig. 2) into the dedicated chamber of the top shell and route its JST cable through the cable channel, as shown in Fig. 5-A1.
2. Mounting of the TSL2561 sensor board (component 3) on its fixing posts in the base shell, ensuring that the active sensing area is centered with respect to the LED board placed in the previous step, as shown in Fig. 5-A2.
3. Mounting of the velocity sensor board (component 2) on the adjacent chamber of the base shell, ensuring that the two LDRs are aligned with the TSL2561 sensor, as shown in Fig. 5-A2.
4. Insertion of the bonded PMMA microfluidic chip (component 4) into the bottom chamber of the base shell, with the inlet and outlet through-holes facing upwards. Verify that the main channel is on top of the velocity sensor LDRS axis and centered above the TSL2561 active area, as shown in Fig. 5-A3.
5. Connection of the three JST cables (LED source, TSL2561, and velocity sensor) to their respective sockets on the Arduino-Zero shield. Mount the shield on top of an Arduino Zero microcontroller and seat the Micro-SD card in the SPI breakout, as shown in Fig. 5-A4.
6. Closing of the assembly by fitting the top cover (component 5) onto the base shell, shielding the optical path from ambient light, as shown in Fig. 5-A4.
7. Connection of the inlet and outlet ports to the syringe pumps and waste reservoir using Tygon tubing (0.4 mm inner diameter). Power the Arduino Zero through the native USB port.

## 6 Operation instructions

The operation of the modular optoelectrical sensor platform is organized into two sequential firmware stages: (i) droplet velocity measurement using the *Velocity Sensor* firmware, and (ii) droplet detection and characterization using the *Droplet Characterization* firmware. The mean droplet velocity $\overset{¯}{V}$ obtained in stage (i) is a required input parameter for the characterization algorithms of stage (ii); therefore, the velocity measurement must be completed before the characterization routine is initiated. Both firmware images were developed in VSCode with the PlatformIO extension targeting the Arduino-Zero board, and each module can be built, flashed, and operated independently provided that the velocity-first dependency is respected.

**Fig. 6:**
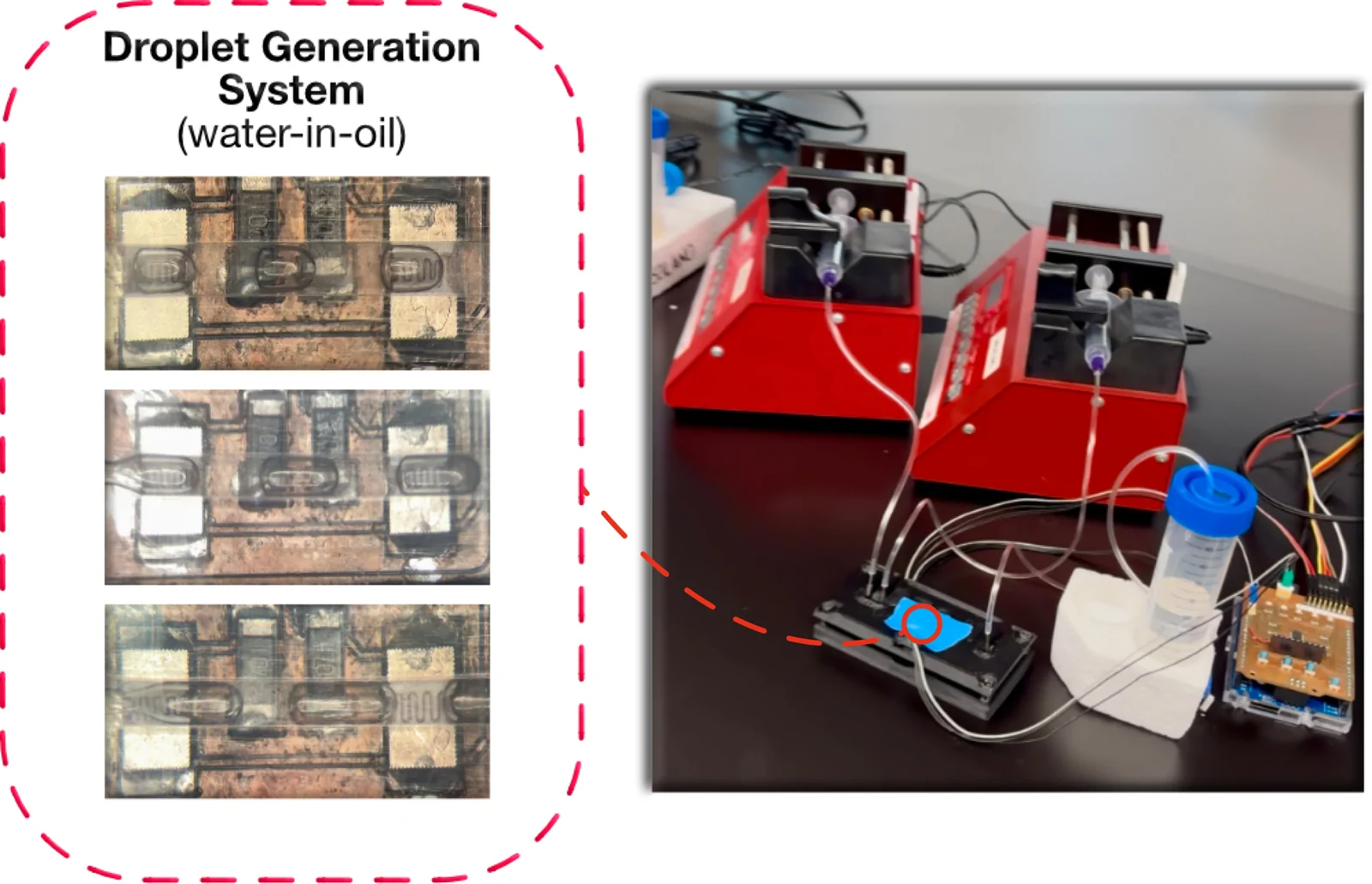
Experimental setup of the modular optoelectrical sensor platform: electronics assembly, microfluidic chip with covers, syringe pump inlets (continuous phase: oil; dispersed phase: water), and waste reservoir outlet. Inset: representative small, medium, and large slug regimes at different oil-to-water flow-rate ratios.

The experimental setup is illustrated in Fig. 6, which shows all assembly and connection steps for the modular sensor system: the electronics boards, microfluidic chip with covers, syringe pump connections (continuous and dispersed phases), and outlet tubing to a waste reservoir. The figure inset illustrates representative droplet generation regimes — small, medium, and large slugs — achieved by adjusting the flow-rate ratio between the continuous phase (oil) and the dispersed phase (water).

### 6.1 Velocity sensor firmware

The *Velocity Sensor* firmware, provided as a PlatformIO project under the firmware/velocity_sensor/ directory, measures the droplet velocity by time-of-flight across the two light-dependent resistors S1 and S2 of the velocity sensor board. The transit time Δt of a single droplet from S1 to S2 is converted to a velocity value through the relation V=d/Δt, where d=9.88mm is the fixed center-to-center separation between the two LDRs. The firmware outputs one velocity reading per computation cycle to the serial monitor; the operator collects multiple readings and averages them to obtain a representative $\overset{¯}{V}$ for the operating flow conditions.

**Fig. 7:**
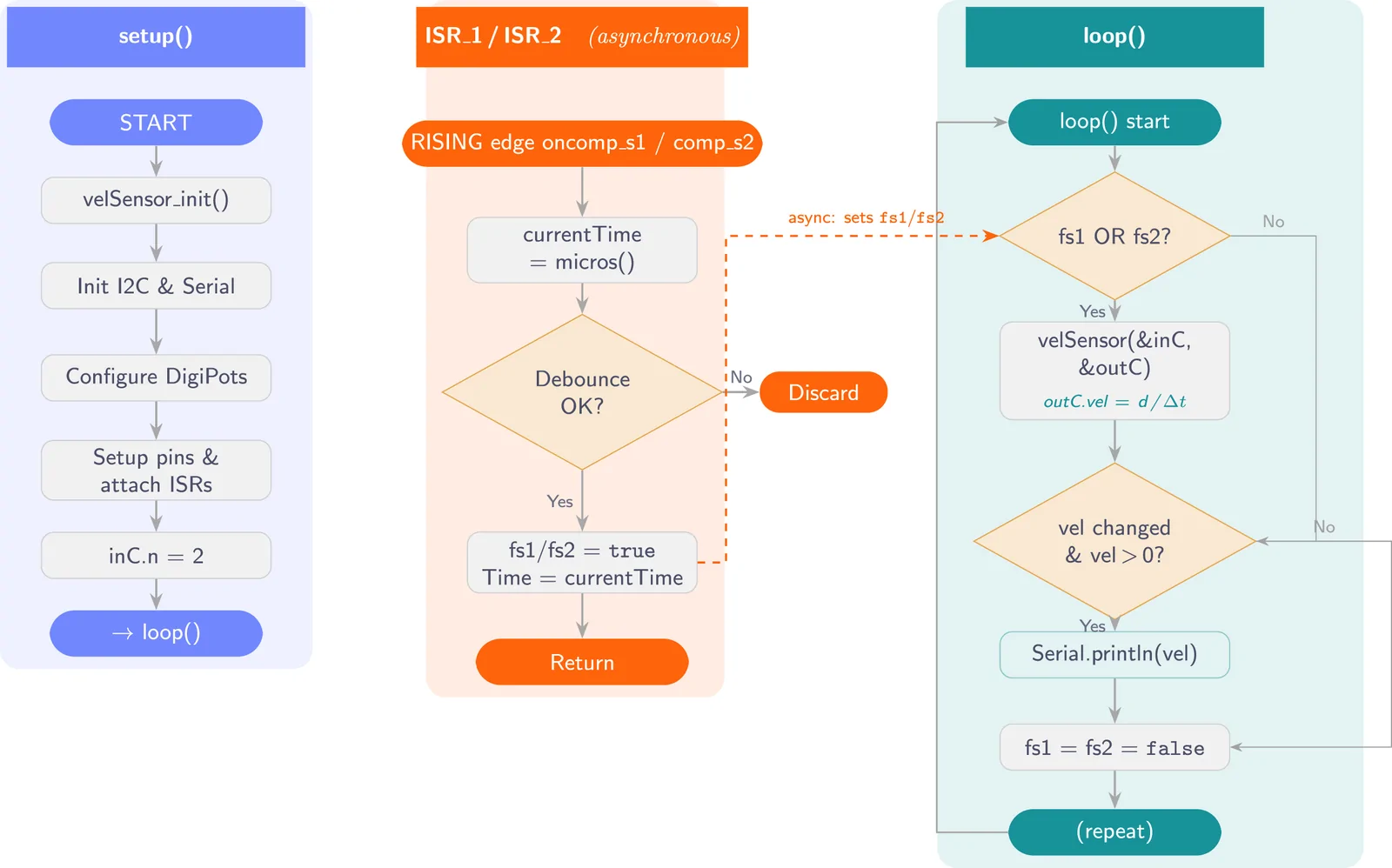
Velocity sensor firmware flowchart: S1/S2 time-of-flight acquisition and velocity computation (V=d/Δt, d=9.88mm). One reading is output per cycle; the operator averages collected serial-monitor values to obtain $\overset{¯}{V}$.

The firmware execution flow is summarized in Fig. 7, in which the three color-coded regions correspond to the configuration, acquisition, and computation stages of the program. The blue region groups the setup() routine, which loads the two digital potentiometers with the wiper values that set the comparator thresholds of sensors S1 and S2, configures the comparator input pins and the status LEDs, opens the serial port, and attaches the two interrupt service routines (ISRs) to the comparator outputs prior to operation.

The orange region describes the acquisition stage. The raw signal of each LDR is a light-intensity-dependent voltage, which the Schmitt-trigger comparator converts into a single digital transition when a droplet enters the corresponding sensing area; the quantity acquired by the microcontroller is therefore not a sampled waveform but the arrival time of that transition. The routines isr_1() and isr_2() are attached to the rising edges of the comparator outputs of sensors S1 and S2, and each routine timestamps its detection with the micros() function. Every candidate edge is validated within the routine by a debounce window applied between consecutive detections of the same sensor, so that invalid transitions caused by signal noise are discarded before the timestamp is accepted.

The green region corresponds to the velSensor() block, a state machine that starts the transit timer on the first accepted S1 detection and stops it on the nth accepted S2 detection. The transit time Δt is obtained as the difference between the two timestamps and the velocity is computed as V=d/Δt; the timer is then reset, and the cycle is repeated for the following droplet.

This interrupt-driven architecture captures the detection instants within the interrupt context rather than at the rate of the main loop, so that the accuracy of Δt is independent of the loop period and of the serial transmission time, while the main loop remains available for LED status indication and serial output. The distinction becomes relevant towards the upper bound of the measurable velocity range, where the transit time between both sensors falls to a few tens of milliseconds.

The droplet-velocity detection firmware is operated as follows.

1. Open the firmware/velocity_sensor/ project in VSCode with the PlatformIO extension installed. Verify that the platformio.ini configuration file targets the zeroUSB board environment and sets the serial monitor baud rate to 38 400 bps.
2. Connect the Arduino-Zero to the host computer via its native USB port. Build and upload the firmware using the PlatformIO *Upload* command, then open the serial monitor at 38 400 bps.
3. Verify the startup banner reported on the serial monitor and record the active configuration parameters: the two digital potentiometer wiper registers (WIPER1 and WIPER2, nominally 76 and 83, respectively) that set the comparator detection thresholds for sensors S1 and S2, and the accumulation parameter n (default n=2) that defines the number of S2 detections averaged per velocity computation cycle.
4. Initiate droplet generation by activating the syringe pumps at the desired flow rates to establish a stable flow-focusing regime in the microfluidic chip.
5. Observe the LED status indicators on the shield to confirm correct sensor operation during droplet flow:
  • The **blue LED** (pin A5) illuminates briefly upon each detection at sensor S1, indicating that the time-of-flight timer has started and the firmware is awaiting the downstream detection at sensor S2.
  • The **yellow LED** (pin 12) illuminates briefly upon each detection at sensor S2, indicating that the timer has stopped and a velocity value is being computed and transmitted.
6. Read the velocity output from the serial monitor. Each valid measurement cycle prints a single floating-point velocity value in $\mathrm{mm}\;s^{-1}$ on a new line, computed as V=d/Δt, where d=9.88mm is the known center-to-center separation between S1 and S2 and Δt is the measured transit time in seconds. Collect as many samples as the experiment requires to obtain a representative mean velocity for the operating flow conditions.
7. If no detections are observed or the velocity readings are erratic, adjust the comparator detection thresholds by modifying the WIPER1 and WIPER2 constants in src/Config.h in increments of 5 and re-uploading the firmware until stable, repeatable detections are obtained for both sensors. Higher wiper values raise the comparator threshold and reduce sensitivity to ambient noise; lower values increase sensitivity but may introduce spurious triggers.
8. Once a stable velocity dataset has been collected, record the mean value $\overset{¯}{V}$ for use as the input parameter of the *Droplet Characterization* firmware described in the following subsection.

### 6.2 Droplet characterization firmware

**Fig. 8:**
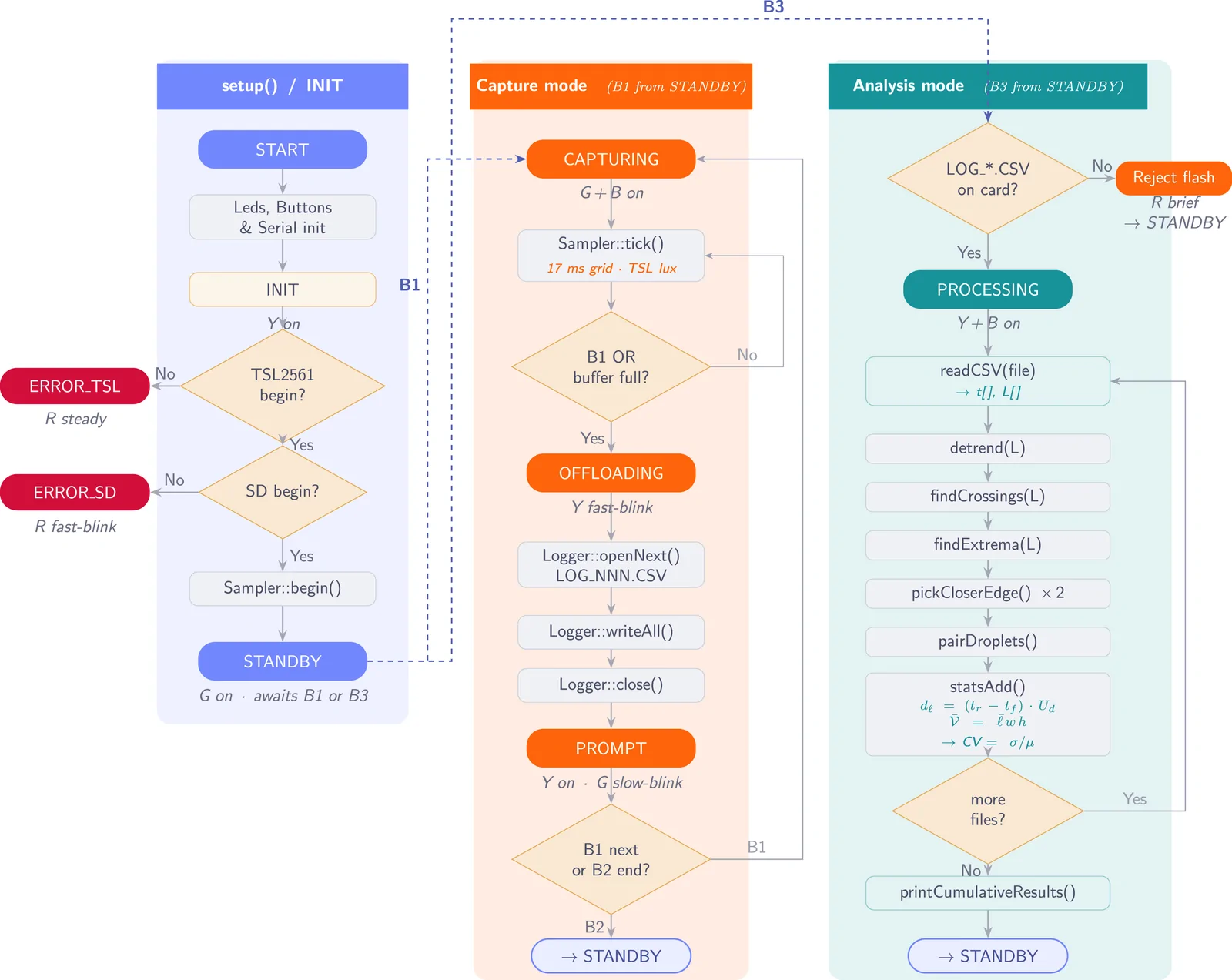
Droplet characterization firmware flowchart: Capture mode (TSL2561 lux sampling at ≈58 Hz to microSD) and Analysis mode (signal detrending, zero-crossing detection, edge pairing, and Welford statistics to report $\overset{¯}{\ell }$, σ, $\overset{¯}{V}$, and CV).

The *Droplet Characterization* firmware, provided as a PlatformIO project under the firmware/optical_flow_sensor/ directory, implements two sequential operating modes on a single firmware image: a *Capture* mode that logs the raw TSL2561 lux signal to the microSD card, and an *Analysis* mode that processes the logged files on-device to report droplet length statistics and the corresponding volume approximation. The mean droplet velocity $\overset{¯}{V}$ obtained from the *Velocity Sensor* firmware is required as a compile-time constant, as detailed below. The firmware execution flow for both modes is summarized in Fig. 8.

The three color-coded regions of Fig. 8 correspond to the initialization, capture, and analysis stages of the program. The blue region groups the setup() routine, which initializes the status LEDs, the push buttons, and the serial port, verifies the presence of the TSL2561 sensor and of the microSD card, and leaves the device in the **Standby** state.

The orange region describes how the raw data is obtained. The droplet stream modulates the light transmitted from the LED source board to the TSL2561, so that each droplet crossing the sensing area produces a transient decrease of the measured illuminance. In the **Capturing** state, the Sampler::tick() routine reads the sensor on a fixed 17 ms grid, set by the 13 ms integration time of the TSL2561 and the associated bus overhead, and stores every reading together with its acquisition time in the RAM buffer. This pair of values is the only quantity retained, and the buffer is transferred without modification to the corresponding LOG_NNN.CSV file during **Offloading**, so that each log file is an unprocessed record of the optical signal.

The green region describes how those files are processed on the device, following the sequence detailed in the *Analysis mode operation* paragraph below. Subtracting the mean of a file removes the baseline transmitted intensity and centers the droplet transitions about zero, so that a falling zero crossing corresponds to the entry of a droplet into the sensing area and the following rising crossing to its exit. Pairing both crossings yields the residence time of each complete droplet, which is converted into a length through the mean velocity $\overset{¯}{V}$ supplied by the previous stage, whereas droplets that were only partially recorded at the beginning or at the end of a file remain unpaired and are discarded.

#### Pre-flash configuration.

Open the file src/Config.h in the firmware/optical_flow_sensor/ project and set the constant UD_MM_PER_S to the mean velocity $\overset{¯}{V}$ recorded in the previous stage, expressed in $\mathrm{mm}\;s^{-1}$. This parameter scales all reported droplet lengths through the relation $\ell =\Delta t\times \overset{¯}{V}$, where Δt is the measured transit time of a droplet across the TSL2561 sensing area. Set the constants CH_WIDTH_MM and CH_HEIGHT_MM to the width w and depth h of the main channel of the installed chip, both 1.40 mm for the flow-focusing chip of Fig. 4; their product is the channel cross-section used for the droplet volume approximation, and it is the only value that changes when the chip is exchanged. Save the file, rebuild the firmware using the PlatformIO *Build* command, and upload the resulting binary to the Arduino-Zero via the native USB port.

#### Startup and error checking.

Upon power-on, the yellow LED illuminates briefly (<1s) while the firmware probes the TSL2561 sensor on the I^2^C bus and the microSD card on the SPI bus. If both peripherals are detected successfully, the yellow LED turns off and the green LED turns on steadily, indicating that the device is in the **Standby** state and ready for user interaction. If the TSL2561 is not detected, the red LED illuminates steadily; if the microSD card is absent or unreadable, the red LED blinks at approximately 5 Hz. In either error condition, the system must be power-cycled after the fault is corrected before operation is resumed.

#### Capture mode operation.

The light-intensity data-logging workflow is initiated from the Standby state as follows.

1. From **Standby** (green LED steady), press button B1 to initiate a capture burst. The green and blue LEDs illuminate simultaneously, indicating that the **Capturing** state is active and the firmware is sampling the TSL2561 sensor at approximately 58 Hz (one sample every 17 ms) into an internal RAM buffer of up to 3 000 samples (≈51s of data).
2. Allow sampling to continue for the desired duration. Capture terminates automatically when the buffer reaches its 3 000-sample capacity, or can be stopped early by pressing B1 again. Both conditions transition the firmware to the **Offloading** state.
3. During **Offloading** (yellow LED fast-blinking at ≈10 Hz), the buffered samples are written to the next available file on the microSD card (auto-named LOG_001.CSV, LOG_002.CSV, and so on, up to LOG_999.CSV). **Do not remove power or the microSD card during this phase**; offloading typically completes within 1–3 s.
4. After offloading completes, the firmware enters the **Prompt** state (yellow LED steady, green LED slow-blinking at ≈1 Hz), in which two options are offered: press B1 to initiate an additional capture burst into a new log file, or press B2 to end the session and return to **Standby**. Repeat steps 1–4 as many times as the experiment requires.

#### Analysis mode operation.

Once all capture sessions have been completed and the device is in **Standby**, the on-device signal-processing pipeline is initiated as follows.

1. Open the serial monitor on the host computer at 115 200 bps to receive the analysis output.
2. Press button B3 from **Standby** to enter the **Processing** state (yellow and blue LEDs on). The firmware reads each LOG_NNN.CSV file stored on the microSD card in sequential numerical order and applies the following pipeline to each file: (i) mean subtraction (detrending) of the raw lux signal, (ii) zero-crossing detection to locate droplet entry and exit edges, (iii) extrema finding and edge refinement to improve crossing-time accuracy, and (iv) edge pairing to identify complete, fully captured droplets. Individual droplet lengths are accumulated across all processed files using Welford’s algorithm to produce numerically stable cross-file statistics.
3. Per-file progress lines are printed to the serial monitor as each file is processed. After the last file is analyzed, the firmware prints the cumulative characterization results and returns automatically to **Standby** (green LED steady). The reported results are:
  • **Total droplets**: cumulative count of fully detected droplets across all log files.
  • **Average length** ($\overset{¯}{\ell }$): mean droplet length in μm, computed as $\overset{¯}{\ell }=\bar{\Delta t}\times \overset{¯}{V}$.
  • **Standard deviation** (σ): sample standard deviation of individual droplet lengths in μm.
  • **Average volume** ($\overset{¯}{V}$): droplet volume approximation in μL, computed as $\overset{¯}{V}=\overset{¯}{\ell }\;w\;h$ from the channel cross-section declared in src/Config.h.
  • **Standard deviation of the volume** ($\sigma_{V}$): the length standard deviation scaled by the same cross-section, $\sigma_{V}=\sigma \;w\;h$.
  • **Coefficient of variation** (CV): monodispersity index defined as $CV=\sigma /\overset{¯}{\ell }\times 100\text{\%}$, reported once because the cross-section scaling leaves it unchanged.
4. The analysis pass is non-destructive and read-only; no file on the microSD card is modified. The pass may be repeated on the same set of log files to verify the results, with each pass producing a fresh cumulative calculation.

### 6.3 Safety

The Arduino-Zero accepts either a regulated 5 V USB supply or an external battery pack, and all sensing and illumination circuits run at 3.3 V. No component dissipates appreciable heat, no part of the assembly moves, and the light source is a pair of low-power indicator LEDs confined within the opaque enclosure, so no eye protection is required. Under these conditions, operation presents no electrical, thermal, mechanical, or optical hazard.

The fluids used in this work, deionized water and paraffin oil, are neither flammable nor toxic; other fluid pairs, in particular volatile solvents or biological samples, must be handled according to their safety data sheet. The inlet and outlet tubing must be seated firmly and the chip must pass the leak test described in the build instructions before it is placed in the base shell, since the sensor boards are housed directly beneath it. Gloves are recommended for filling the syringes, connecting the tubing, and installing the chip; they should be removed before device operation, and power must be disconnected before any spilled liquid is wiped from the enclosure.

## 7 Validation and characterization

This section demonstrates and characterizes the performance of the assembled platform across its four measured parameters (droplet length, volume, velocity, and monodispersity), directly on chip and with embedded post-processing. All data reported below were acquired with the hardware and firmware described in the preceding sections, operated under the workflow detailed in Fig. 8. The characterization is organized into two complementary experiments: a droplet detection and characterization study across two flow-focusing chip configurations with different channel widths, and an on-chip velocity measurement study that also quantifies the relationship between droplet velocity and monodispersity across a range of flow-rate ratios. The underlying physical models and a fuller fluid-dynamics analysis are reported in the authors’ companion studies [14], [20].

### 7.1 Droplet detection and characterization across channel widths

The TSL2561 sensing stage is validated on two flow-focusing chip configurations: a primary 1.4 mm wide channel and a reduced 700 μm wide channel [14]. Both chips are operated with deionized water as the dispersed phase ($\rho =1000\;\mathrm{kg}\;m^{-3}$) and Miglyol 829 N as the continuous phase ($\rho =1000\;\mathrm{kg}\;m^{-3}$, μ=230mPas, $\gamma =0.05\;N\;m^{-1}$), corresponding to a capillary number of Ca=0.0032 in both configurations.

For the 1.4 mm channel, six independent experiments are conducted at matched flow rates of $Q_{c}=Q_{d}=37.632\;\mathrm{mL}\;h^{-1}$, yielding a population of 119 droplets generated at a rate of approximately 1.8 droplets s^−1^. The on-chip sensor reports a mean droplet length of 4.006±0.026mm, and an average relative error of 2.06% with respect to references values processed with Fiji, with per-experiment relative errors ranging from 0.37% to 7.64%. The monodispersity (CV(%)) found across the six experiments ranges from 6.29% to 10.58%, with a mean of 8.27%.

The 700 μm configuration is operated at $Q_{c}=Q_{d}=1.085\;\mathrm{mL}\;h^{-1}$ to preserve the same capillary number, and the on-chip sensor reports a mean droplet length of 1617.4±38.08μm over 35 droplets acquired in a single experiment, a mean relative error of 2.60% with respect to the reference, and a monodispersity of 2.35%. The reduction in CV from 8.27% on the 1.4 mm channel to 2.35% on the 700 μm channel is attributable to the refined inlet and outlet interfaces of the smaller chip and confirms that the sensing stage transfers to a different channel width without recalibration, by interchanging only the microfluidic module.

The main improvement presented in this work, over the configuration reported in [14] is the migration of the droplet-length signal-processing pipeline from an off-chip MATLAB script to on-device firmware running on the Arduino-Zero microcontroller. The pipeline retains the original sequence of mean subtraction of the raw lux signal, zero-crossing edge detection, edge pairing, and droplet-length statistics, and is invoked through the *Analysis* mode of the *Droplet Characterization* firmware described in Fig. 8, which produces the cumulative droplet count, the mean length $\overset{¯}{\ell }$, the standard deviation σ, the droplet volume approximation $\overset{¯}{V}$, and the coefficient of variation CV directly on the device. The volume approximation is obtained from the mean length and the main-channel cross-section declared in the firmware, yielding $\overset{¯}{V}=7.85\;\mu L$ for the 1.4 mm configuration and 0.79μL for the 700 μm one. This migration removes the host-side post-processing dependency present in the prior validation and renders the characterization stage fully self-contained.

### 7.2 On-chip velocity measurement and velocity versus monodispersity

The droplet velocity sensing stage is validated using the LDR-array configuration of the *Velocity Sensor* firmware over six flow-rate ratios $\phi =Q_{d}/Q_{c}$ spanning 0.5 to 2.0, while the continuous-phase flow rate is held constant at $Q_{c}=14.59\;\mathrm{mL}\;h^{-1}$ [20]. The experiments employ deionized water as the dispersed phase (η=1.0mPas, $\rho =998\;\mathrm{kg}\;m^{-3}$) and paraffin oil as the continuous phase (η=145mPas, $\rho \approx 858\;\mathrm{kg}\;m^{-3}$), at a capillary number of Ca=0.006 and surface tension $\gamma =0.05\;N\;m^{-1}$. A total of 797 droplets are analyzed and compared against a high-speed imaging reference processed in Fiji. Across the six operating points, the on-chip sensor reports droplet velocities in the range 3.7 to 7.4 $\mathrm{mm}\;s^{-1}$ and droplet lengths in the range 1.9 to 5.0 mm, with an average velocity deviation of 5.32% with respect to the optical reference. Per-condition deviations span 0.74% at ϕ=1.50 to 12.07% at ϕ=0.50.

The on-chip velocity and droplet-length data enables the quantification of two design-relevant relationships. For these experiments, the mean droplet velocity scaled linearly with the inverse flow-rate ratio, as described by Eq. (1), where V is expressed in $\mathrm{mm}\;s^{-1}$ and the fit yields a coefficient of determination $R^{2}=0.989$. The regression is computed on n=6 operating points, each obtained from between 116 and 142 droplets. That number of points supports the reciprocal trend but does not fix the two coefficients precisely, so the expression describes the measured range rather than predicting behavior outside it.

(1) $$V=\frac{2.51}{\phi }+2.45$$

Additionally, the assessment of monodispersity resulted in an inverse power-law dependence on the mean velocity, as described by Eq. (2), with prefactor C=1.74, threshold velocity $a=2.49\;\mathrm{mm}\;s^{-1}$, and exponent k=1.14. The fit yields $R^{2}=0.912$ over the same n=6 operating points, and the measured CV values span 2.1% to 10%. The three parameters rest on the same six points and are therefore preliminary estimates, supported only within the measured velocity range of 3.7 to 7.4 $\mathrm{mm}\;s^{-1}$.

(2) $$CV=1.74\;{\left(V-2.49\right)}^{-1.14}$$

Both empirical relationships are specific to the conditions employed in these on-chip experiments, namely the water-in-paraffin-oil system, the 1.4 mm flow-focusing geometry, the fixed continuous-phase flow rate, and the flow-rate ratio range ϕ∈[0.5,2.0]. Their applicability to other fluid combinations or operating regimes has not been established and would require independent experimental validation.

Under the conditions tested, Eq. (2) provides a practical operating envelope for the platform: monodispersity degrades as droplet velocity increases, so flow conditions that maximize droplet uniformity correspond to lower velocity regimes near the threshold of the model.

### 7.3 Capabilities and limitations

The validation studies summarized above define the present operating envelope of the platform. The on-device firmware reports droplet length, volume, velocity, and monodispersity directly, with droplet-length and monodispersity pipelines executed locally on the Arduino-Zero and eliminating the post-processing dependency carried over from [14]. The same optical sensing stage transfers across flow-focusing geometries of 1.4 mm and 700 μm without recalibration, requiring only the replacement of the microfluidic chip. Droplet velocity is recovered over the validated range of 3.7 to 7.4 $\mathrm{mm}\;s^{-1}$ with an average deviation of 5.32% from an external video reference values.

The upper bound of the measurable velocity range is governed by the response dynamics of the LDR array: the measured fall time of $\tau_{\mathrm{fall}}=36.75\pm 2.76\;\mathrm{ms}$ imposes a detection delay of approximately 11.5 ms, which translates into a maximum resolvable velocity of 522 $\mathrm{mm}\;s^{-1}$ for elongated droplets and approximately 87 $\mathrm{mm}\;s^{-1}$ at the minimum detectable droplet length of 1 mm [20]. Applications demanding higher droplet velocities or sampling rates beyond the LDR operating range can substitute the light-dependent resistors with photodiodes equipped with transimpedance amplifiers, which extend the measurable range at the cost of increased circuit complexity, fabrication effort, and component cost [20]. The modular architecture supports reuse of the sensing electronics across different channel geometries provided that the chip is optically translucent and conforms to the 75 by 25 mm microscope-slide footprint; the alignment enclosure must be in place during operation to prevent ambient illumination from biasing the TSL2561 and LDR signals; and the *Droplet Characterization* firmware requires a prior mean velocity $\overset{¯}{V}$ supplied by the *Velocity Sensor* firmware, which fixes the order of operation between the two sensing stages.

Measurement is therefore performed in two stages rather than concurrently, a limitation of the present firmware implementation and not of the sensing architecture. Concurrent operation remains possible in principle, either on a microcontroller able to service both acquisition tasks at once or through an initial calibration routine that supplies the velocity to the characterization pipeline at run time, and is left for future work.

The characterization stage is intended for flow-focusing generation devices, in which the velocity of successive droplets is expected to remain close to its mean and the single value $\overset{¯}{V}$ declared in the firmware is representative of the whole population. Flows in which the droplet velocity varies can be analyzed through the per-droplet velocity output of the *Velocity Sensor* firmware before the characterization stage is run. Both firmware stages must be operated under identical flow conditions, since a change between them alters the generated droplets themselves and not only the value of $\overset{¯}{V}$. Extending the firmware with an automatic stability check that warns the operator when droplet generation departs from the expected population is left for future work.

## CRediT authorship contribution statement

**Daniel Solano:** Writing – review & editing, Writing – original draft, Visualization, Validation, Resources, Project administration, Methodology, Investigation, Formal analysis, Data curation, Conceptualization. **José Isabel Gómez-Quiñones:** Writing – review & editing. **Sergio Camacho-Leon:** Writing – review & editing, Supervision, Investigation, Formal analysis.

## Declaration of competing interest

The authors declare that they have no known competing financial interests or personal relationships that could have appeared to influence the work reported in this paper.
